# Predictive models for small-for-gestational-age births in women exposed to pesticides before pregnancy based on multiple machine learning algorithms

**DOI:** 10.3389/fpubh.2022.940182

**Published:** 2022-08-08

**Authors:** Xi Bai, Zhibo Zhou, Mingliang Su, Yansheng Li, Liuqing Yang, Kejia Liu, Hongbo Yang, Huijuan Zhu, Shi Chen, Hui Pan

**Affiliations:** ^1^Key Laboratory of Endocrinology of National Health Commission, Department of Endocrinology, State Key Laboratory of Complex Severe and Rare Diseases, Peking Union Medical College Hospital, Chinese Academy of Medical Science and Peking Union Medical College, Beijing, China; ^2^DHC Mediway Technology Co., Ltd, Beijing, China

**Keywords:** small for gestational age, exposure to pesticides, machine learning, prediction, environmental pollution

## Abstract

**Background:**

The association between prenatal pesticide exposures and a higher incidence of small-for-gestational-age (SGA) births has been reported. No prediction model has been developed for SGA neonates in pregnant women exposed to pesticides prior to pregnancy.

**Methods:**

A retrospective cohort study was conducted using information from the National Free Preconception Health Examination Project between 2010 and 2012. A development set (*n* = 606) and a validation set (*n* = 151) of the dataset were split at random. Traditional logistic regression (LR) method and six machine learning classifiers were used to develop prediction models for SGA neonates. The Shapley Additive Explanation (SHAP) model was applied to determine the most influential variables that contributed to the outcome of the prediction.

**Results:**

757 neonates in total were analyzed. SGA occurred in 12.9% (*n* = 98) of cases overall. With an area under the receiver-operating-characteristic curve (AUC) of 0.855 [95% confidence interval (CI): 0.752–0.959], the model based on category boosting (CatBoost) algorithm obtained the best performance in the validation set. With the exception of the LR model (AUC: 0.691, 95% CI: 0.554–0.828), all models had good AUCs. Using recursive feature elimination (RFE) approach to perform the feature selection, we included 15 variables in the final model based on CatBoost classifier, achieving the AUC of 0.811 (95% CI: 0.675–0.947).

**Conclusions:**

Machine learning algorithms can develop satisfactory tools for SGA prediction in mothers exposed to pesticides prior to pregnancy, which might become a tool to predict SGA neonates in the high-risk population.

## Introduction

Small-for-gestational-age (SGA) newborns are defined as birth weight below the 10^th^ percentile of gestational age standards based on a specific population (1, 2). Infants born SGA are at increased risk for perinatal morbidity and mortality (3, 4). Unrecognized SGA before birth is a major risk factor for stillbirth (5), highlighting the importance of prenatal prediction of SGA because it permits closer surveillance and timely delivery to decrease adverse birth outcomes. In fact, the risk of stillbirth can be significantly decreased, even by four times, if SGA newborns can be detected before delivery (6).

Adverse environmental factors have been related to a reduction in birth weight (7–9). The association between prenatal pesticide exposures and reduced birth weight has been reported in human studies. Women exposed to the highest quartile of 4-nitrophenol were at increased risk of delivering SGA neonates, with a relative risk of 3.81 (1.10, 13.21) (10). Also, a case-control study in India demonstrated that prenatal exposure to some organochlorine pesticides might impair fetal anthropometric development, reducing birth weight, length, head circumference, and chest circumference (11). Additionally, it has been reported that pyrethroids exposure was associated with a slower rate of fetal development at birth with SGA (12). Fetuses could appear to be more susceptible to pesticide residues than adults because of their rapid growth, developing organ systems, and immature metabolic pathways (13). However, there are no studies developing a tool for SGA prediction in mothers exposed to pesticides prior to pregnancy.

Because of the inherent constraints of not incorporating the underlying interactions between variables, risk prediction models based on traditional statistical approaches have a negative impact on their use and efficacy in big datasets with numerous features (14, 15). However, machine learning (ML) techniques, which could handle complicated relationships and optimize prediction performance from complicated dataset, can overcome these restrictions (16, 17). As for predicting the risk of SGA, in a few studies, ML classifiers were used to develop SGA prediction tools in the overall population (18–22). Unfortunately, the prediction tools did not perform well, with a maximum area under the receiver operating characteristic (ROC) curve (AUC) of 0.7+. Besides, several paternal features and maternal exposure to PM2.5 in pregnancy have been reported as independent risk factors for SGA neonates (23–26). Despite the fact that these associations have been confirmed, the combination of them has not previously been included in prediction tools.

In our study, based on a prospective cohort study from the National Free Preconception Health Examination Project (NFPHEP) in China, multiple ML algorithms were applied to establish and validate prediction models for SGA neonates in mothers exposed to pesticides in a living or working environment prior to pregnancy.

## Materials and methods

### Dataset source

Data were obtained from the NFPHEP, a three-year project initiated by the National Health Commission of the People's Republic of China and conducted in 220 counties across 31 provinces or municipalities from 1 January 2010 to 31 December 2012. The general design and implementation of the NFPHEP were reported in previous publications (27–29). The goal of the NFPHEP was to explore risk factors for poor birth and to promote the health of mothers and their babies. The National Quality Inspection Center for Family Planning Techniques performed the quality control on all data before uploading them to a nationwide electronic data collecting system. The Institutional Review Committee of the National Research Institute for Family Planning in Beijing, China, approved this study (protocol code 2017101702), and all participants gave their informed consent.

### Study participants and features

This study included all singleton neonates with a full birth record and a gestational age > 24 weeks, after which cases whose mothers had self-reported exposure to pesticides in their living or working environment prior to pregnancy were selected. The final analysis comprised 757 newborns after records with incomplete data and extreme characteristic values were removed.

A pre-pregnancy checkup was performed, as well as a pregnancy and postpartum follow-up. 142 features about the parental demographic factors, style of living, family medical history, existing health issues, laboratory tests, and newborn birth data were obtained by face-to-face inquiry and assessment conducted by experienced and certified personnel. Specifically, the sociodemographic features including pregnancy history, disease history, family history, medication status, living habits, diet and nutrition status, occupational status, working and living environment features, social-psychological features, and interpersonal relationships of the participants were obtained through medical history inquiry. Height, body mass index, blood pressure, heart rate, thyroid palpation, cardiopulmonary auscultation, abdominal palpation, limb spinal examination, and reproductive system examination were obtained by physical examination. Blood routine, urine routine, vaginal secretions, blood type, blood sugar, liver function, kidney function, thyroid function, hepatitis B test, rubella virus, cytomegalovirus, toxoplasma, and treponema pallidum screening were obtained through laboratory tests. The Chinese Center for Disease Control and Prevention reported PM2.5 values for all counties included, based on a historical PM2.5 estimate hindcast model created by satellite-retrieved aerosol optical depth (30). Based on Chinese Neonatal Network, SGA was defined as neonates having a birth weight below the 10^th^ percentile for the gestational age and sex (31).

### Study design

**Figure 1:**
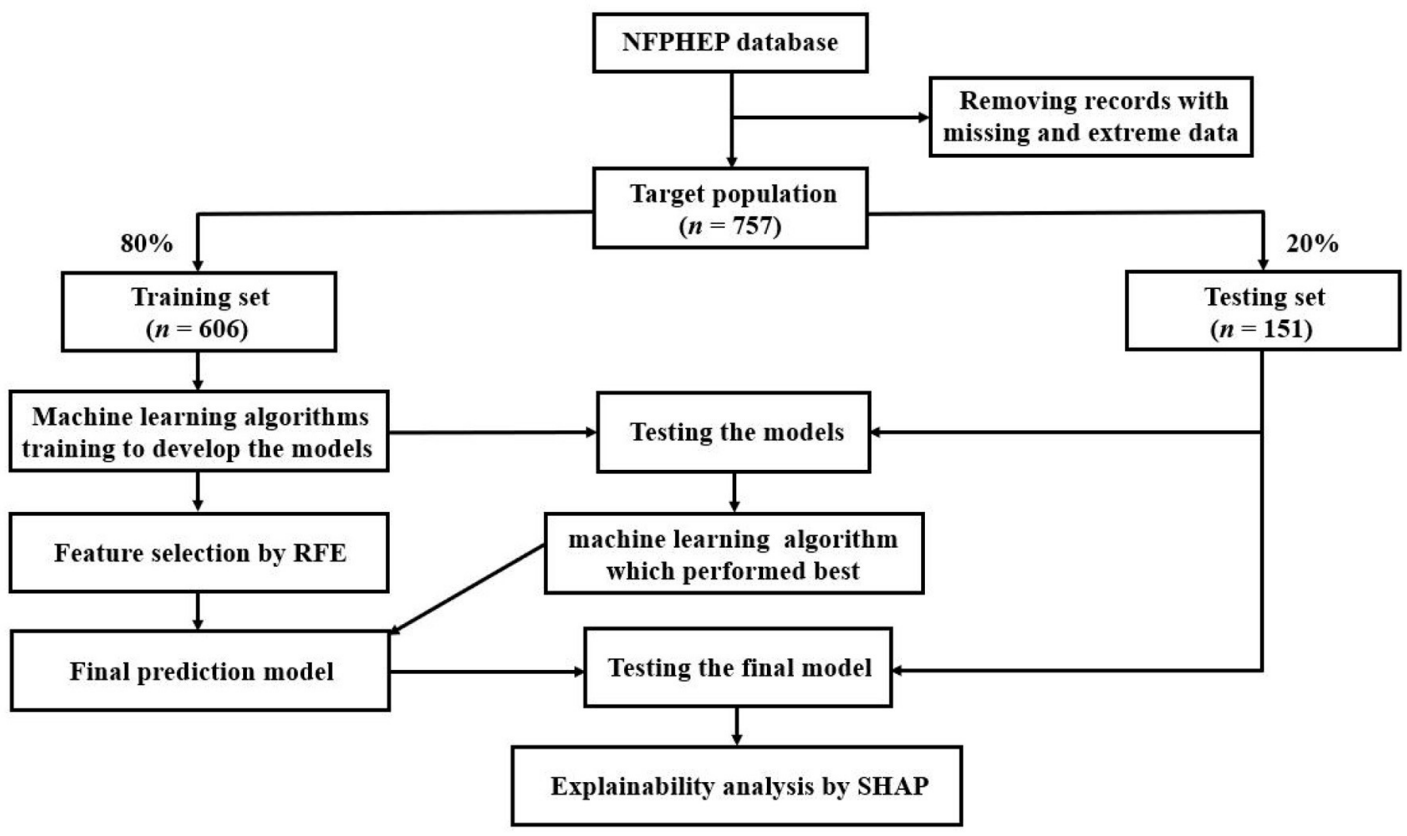
The overall process of data extraction, training, and testing. NFPHEP, National Free Preconception Health Examination Project; RFE, recursive feature elimination; SHAP, Shapley Additive Explanation.

Figure 1 depicts the data processing flow. Python (version 3.8.5) was used to perform all of the analyses. For the generation and testing of ML prediction models, the dataset was randomly divided into a training set (80%, *n* = 606) and a testing set (20%, *n* = 151). Initially, ML algorithms included 142 related features (Supplementary Table S1) as candidate predictor variables. Seven ML classifiers were used to establish prediction models in this study. AUC, sensitivity, specificity, positive predictive value (PPV), and negative predictive value (NPV) were used to assess the performances of the seven ML algorithms. The results of the AUC metric were used as the major parameter for evaluating the efficacy of the ML algorithms.

The category boosting (CatBoost), gradient boosting decision tree (GBDT), and extreme gradient boosting (XGBoost) algorithms were chosen for the final prediction model since they were the top three performing algorithms. To lower the computational cost of modeling, recursive feature elimination (RFE) was used to select 15 variables that contributed significantly to the outcome from 142 variables, using CatBoost, GBDT, and XGBoost as the estimators, respectively. The efficacy of the RFE method has been confirmed in a variety of medical studies (32–35). The 15 most important features were chosen using a 5-fold cross-validation method. These 15 features were added to prediction models and the model which performed best among the three algorithms was chosen as the final prediction model. The tuning of the hyperparameters utilized grid search, and the used hyperparameters of the most effective ML classifier (CatBoost) were learning rate = [0.001, 0.005, 0.01, 0.05, 0.1, 0.2, 0.3, 0.4, 0.5], max depth = [2, 3, 4, 5, 6, 7, 8], l2_leaf_reg = range (1, 50, 1), max leaves = range (1, 50, 1). The features of the final model's hyperparameter tunning were learning rate = 0.01, depth = 5, l2_leaf_reg = 40, loss function = Logloss, eval metric = Accuracy, grow policy = SymmetricTree, model size reg = 0.5, max leaves = 32, random state = 0.

In addition, we used the Shapley Additive Explanation (SHAP) method to apply the *post hoc* explainability on the prediction models based on CatBoost, GBDT and XGBoost classifiers, in order to interpret the impact of variables on the prediction outcome. SHAP uses game theory for evaluating the impact of specific input variables to the outcome of a certain model (36). Moreover, decision curve analysis (DCA) was applied to evaluate the net benefit of the prediction models based on CatBoost, GBDT and XGBoost algorithms.

### ML algorithms

A traditional logistic regression (LR) approach and six widely used ML classifiers, including random forest (RF), GBDT, XGBoost, light gradient boosting machine (LGBM), CatBoost, and support vector machine (SVM), were used in this study for data modeling. All these classifiers are the most prevalent supervised ML approaches handling categorization problems. Utilizing a sigmoid function to calculate logistic transformation of the likelihood, the LR method is applied to estimate the likelihood of the binary dependent variable (37). SVM aims to generate a hyperplane. SVM's primary purpose is to optimize the distance between each class's nearest points, known as support vectors (38, 39). RF is an ensemble classification process which uses majority voting to aggregate multiple decision trees (40, 41). GBDT is built on decision tree ensembles and is known for its precision, effectiveness, and interpretability. The residue between the current prediction and the ground truth is matched by a new decision tree that is trained for each step (42). LGBM uses a histogram to aggregate gradient information, which greatly increases the training efficiency (43). XGBoost is a high-speed, high-performance distributed gradient boosting library. It makes use of the second-order gradient, which improves the approximation greedy search, parallel learning, and hyperparameters (44). CatBoost provides a novel categorical feature handling approach that can address gradient bias and prediction shift (45).

### Statistical analyses

The Chi-square or Fisher's exact test was used to compare categorical features that were expressed as numbers (%). The two-tailed Student's *t*-test was used to compare continuous features that had a normal distribution, expressed as mean [standard deviation (SD)]; otherwise, the median (interquartile range [IQR]) and Wilcoxon Mann-Whitney U test were applied. Models' AUC, sensitivity, specificity, PPV, and NPV were evaluated. The performances of the ML classifiers were assessed by the AUCs in the development and validation sets. Statistical significance was defined as a two-sided *p*-value < 0.05. All statistical analyses were performed using Python (version 3.8.5).

## Results

### Baseline characteristics

From 1 January 2010, to 31 December 2012, the NFPHEP database recorded 757 neonates whose mothers were exposed to pesticides prior to pregnancy. There were 98 SGA births (12.9%) among the 757 neonates. Table 1 displays the demographic features of the study subjects. The findings of comparing the 142 candidate features for predictors in the database are listed in Supplementary Table S1. Overall, the neonates in the cohort had a median gestational age of 39.0 weeks (IQR: 39.0–40.0). SGA neonates had a significantly lower birth weight [2.5 kg (2.2–2.7)] than non-SGA neonates [3.5 kg (3.2–3.7)]. In comparison to the non-SGA group, significant lower paternal height was observed in the SGA group [170.0 cm (165.0–172.0) vs. 170.0 cm (167.0–173.0)]. In addition, proportion of fathers who did not quit smoking during pregnancy in the SGA group was significantly higher than that in the non-SGA group (25.5% vs. 14.0%). Moreover, the proportion of fathers with severe interpersonal pressure in the SGA group was significantly higher than that in the non-SGA group (8.2% vs. 3.2%).

**Table 1:** Demographic characteristics of the subjects by the status of small for gestational age (SGA).

**Parameters**	**Overall** **(*n* = 757)**	**Not SGA** **(*n* = 659)**	**SGA** **(*n* = 98)**	***P* value**
Gestational at birth, week	39.0 (39.0–40.0)	39.0 (39.0–40.0)	39.5 (39.0–40.0)	0.168
Male gender	384.0 (50.7%)	334.0 (50.7%)	50.0 (51.0%)	0.963
Birth weight, kg	3.4 (3.1–3.6)	3.5 (3.2–3.7)	2.5 (2.2–2.7)	<0.001
Maternal age, year	26.0 (23.0–29.0)	26.0 (23.0–29.0)	27.0 (23.0–30.0)	0.487
Paternal age, year	28.0 (24.0–32.0)	28.0 (24.0–32.0)	28.0 (24.3–32.0)	0.392
Maternal height, cm	158.8 ± 5.3	158.9 ± 5.3	157.8 ± 5.7	0.055
Paternal height, cm	170.0 (167.0–173.0)	170.0 (167.0–173.0)	170.0 (165.0–172.0)	0.046
Maternal BMI, kg/m2	21.6 (19.8–23.7)	21.6 (20.0–23.7)	21.3 (19.5–23.5)	0.229
Paternal BMI, kg/m2	22.2 (20.8–24.1)	22.2 (20.8–23.9)	22.0 (20.6–24.5)	0.447
Maternal education level				
Below junior high school	714.0 (94.3%)	619.0 (93.9%)	95.0 (96.9%)	0.442
Senior high school	39.0 (5.2%)	36.0 (5.5%)	3.0 (3.1%)	
Bachelor's degrees and above	4.0 (0.5%)	4.0 (0.6%)	0.0 (0.0%)	
Paternal education level				
Below junior high school	700.0 (92.5%)	608.0 (92.3%)	92.0 (93.9%)	0.838
Senior high school	49.0 (6.5%)	44.0 (6.7%)	5.0 (5.1%)	
Bachelor's degrees and above	8.0 (1.0%)	7.0 (1.0%)	1.0 (1.0%)	
Paternal smoking status				
Quitting smoking	486.0 (64.2%)	429.0 (65.1%)	57.0 (58.2%)	0.012
Reduced smoking	154.0 (20.3%)	138.0 (20.9%)	16.0 (16.3%)	
The same or increased smoking	117.0 (15.5%)	92.0 (14.0%)	25.0 (25.5%)	
Maternal interpersonal pressure				
None	635.0 (83.9%)	557.0 (84.5%)	78.0 (79.6%)	0.380
Mild	98.0 (12.9%)	81.0 (12.3%)	17.0 (17.3%)	
Severe	24.0 (3.2%)	21.0 (3.2%)	3.0 (3.1%)	
Paternal interpersonal pressure				
None	632.0 (83.5%)	556.0 (84.4%)	76.0 (77.5%)	0.045
Mild	96.0 (12.7%)	82.0 (12.4%)	14.0 (14.3%)	
Severe	29.0 (3.8%)	21.0 (3.2%)	8.0 (8.2%)	

BMI, body mass index. Data are presented as median (interquartile range), mean (standard deviation) or number (%).

### Performance evaluation of classification models

**Figure 2:**
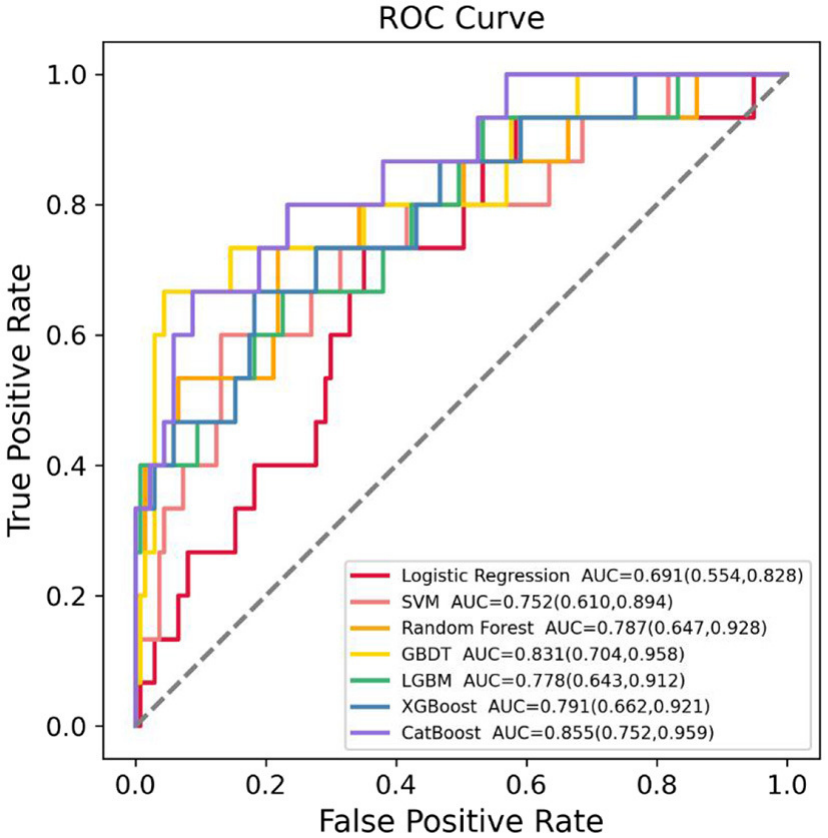
Receiver operating characteristic (ROC) curves of the seven machine learning (ML) models in predicting small for gestational age (SGA) in the testing dataset. SVM, support vector machine; GBDT, gradient boosting decision tree; LGBM, light gradient boosting machine; XGBoost, extreme gradient boosting; CatBoost, category boosting.

The training dataset (*n* = 606) was used to develop the models based on LR, RF, GBDT, XGBoost, LGBM, CatBoost, and SVM algorithms, and the testing dataset (*n* = 151) was used to evaluate their SGA prediction performances. The ROC curve assessment of ML classifiers in the validation set is shown in Figure 2. Overall, the CatBoost model achieved the top AUC value in the testing set, with an AUC of 0.855 [95% confidence interval (CI): 0.752–0.959]. For SGA prediction, all models had a acceptable AUC: CatBoost (AUC: 0.855, 95% CI: 0.752–0.959), GBDT (AUC: 0.831, 95% CI: 0.704–0.958), XGBoost (AUC: 0.791, 95% CI: 0.662–0.921), RF (AUC: 0.787, 95% CI: 0.647–0.928), LGBM (AUC: 0.778, 95% CI: 0.643–0.912), and SVM (AUC: 0.752, 95% CI: 0.610–0.894), with the exception of LR (AUC: 0.691, 95% CI: 0.554–0.828). Furthermore, Table 2 includes the AUCs in the development and validation sets, as well as sensitivity, specificity, PPV, and NPV of each model. Model sensitivity, specificity, PPV, and NPV varied from 0.600 to 0.733, 0.650 to 0.956, 0.186 to 0.625, and 0.952 to 0.964, respectively.

**Table 2:** Performance of models by different algorithms in predicting small for gestational age (SGA) neonates.

**Model**	**AUC**	**AUC**	**Sensitivity**	**Specificity**	**PPV**	**NPV**
	**Training**	**Testing**	**Testing**	**Testing**	**Testing**	**Testing**
LR	0.841	0.691	0.733	0.650	0.186	0.957
SVM	0.763	0.752	0.600	0.869	0.333	0.952
RF	0.943	0.787	0.733	0.781	0.268	0.964
GBDT	0.997	0.831	0.667	0.956	0.625	0.963
XGBoost	0.992	0.791	0.667	0.818	0.286	0.957
LGBM	0.994	0.778	0.667	0.774	0.244	0.955
CatBoost	0.991	0.855	0.667	0.912	0.455	0.962

AUC, area under the receiver-operating-characteristic curve; PPV, positive predictive value; NPV, negative predictive value; LR, logistic regression; SVM, support vector machine; RF, random forest; GBDT, gradient boosting decision tree; XGBoost, extreme gradient boosting; LGBM, light gradient boosting machine; CatBoost, category boosting.

### Feature selection and final prediction model

**Figure 3:**
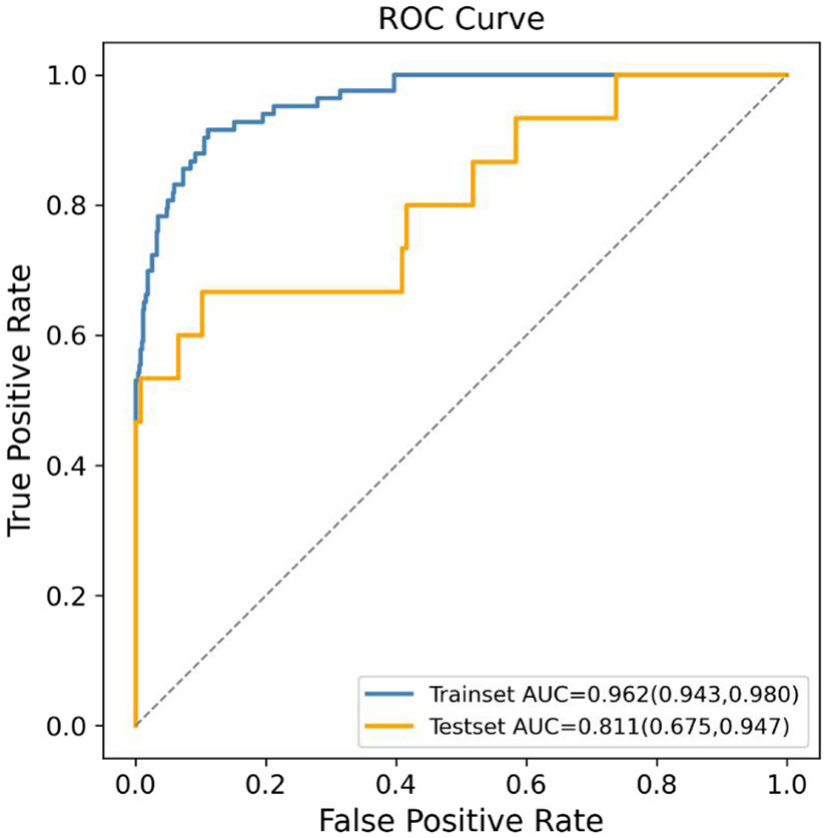
Receiver operating characteristic (ROC) curves of the final machine learning (ML) model using CatBoost algorithm generated after recursive feature elimination (RFE) in predicting small for gestational age (SGA).

The RFE approach was applied to choose 15 features that contributed significantly to the prediction outcome from the 142 features to lower the modeling's computational expense. CatBoost, GBDT, and XGBoost classifiers were chosen as the estimators for RFE since they were the top three performing algorithms. After selecting 15 features using these three algorithms to model, respectively, the AUC values in the testing set of the models based on CatBoost, GBDT, and XGBoost were 0.811 (95% CI: 0.675–0.947), 0.803 (95% CI: 0.665–0.942), and 0.789 (95% CI: 0.643–0.935), respectively. Therefore, the CatBoost model, which achieved the highest AUC result in the comparison of the three models, was chosen as the final prediction model. The 15 variables in the final model were maternal blood type, paternal blood type, maternal exposure to PM2.5 during the late pregnancy, maternal alanine aminotransferase (ALT) prior to pregnancy, paternal smoking status in the first trimester, maternal folacin intake, maternal thyroid-stimulating hormone (TSH) prior to pregnancy, maternal economic pressure prior to pregnancy, paternal life/work stress prior to pregnancy, paternal ALT prior to pregnancy, maternal age, maternal secondhand smoking prior to pregnancy, paternal economic pressure prior to pregnancy, paternal secondhand smoking prior to pregnancy, and maternal contraception prior to pregnancy. The final prediction model's ROC curve results in the development and validation set are shown in Figure 3. The AUC results in the development and validation dataset, sensitivity, specificity, PPV, and NPV in the validation dataset were 0.962 (95% CI: 0.943–0.980), 0.811 (95% CI: 0.675–0.947), 0.667, 0.898, 0.417 and 0.961, respectively, demonstrating the effectiveness of the utilized ML algorithm and feature selection method. Additionally, the DCA result demonstrated the benefit of the final ML model for the prediction of SGA newborns (Supplementary Figure S1). Moreover, the ROC curves, feature selection results, and DCA results of the GBDT and XGBoost models are shown in Supplementary Figure S1 and Supplementary Figures S3–6. Most of the features in the final CatBoost prediction model, including maternal blood type, paternal blood type, maternal exposure to PM2.5 during the late pregnancy, paternal smoking status in the first trimester, maternal ALT, maternal TSH, paternal ALT, maternal economic pressure and paternal economic pressure prior to pregnancy, also contributed significantly in the GBDT and XGBoost model.

### *Post-hoc* interpretation results

**Figure 4:**
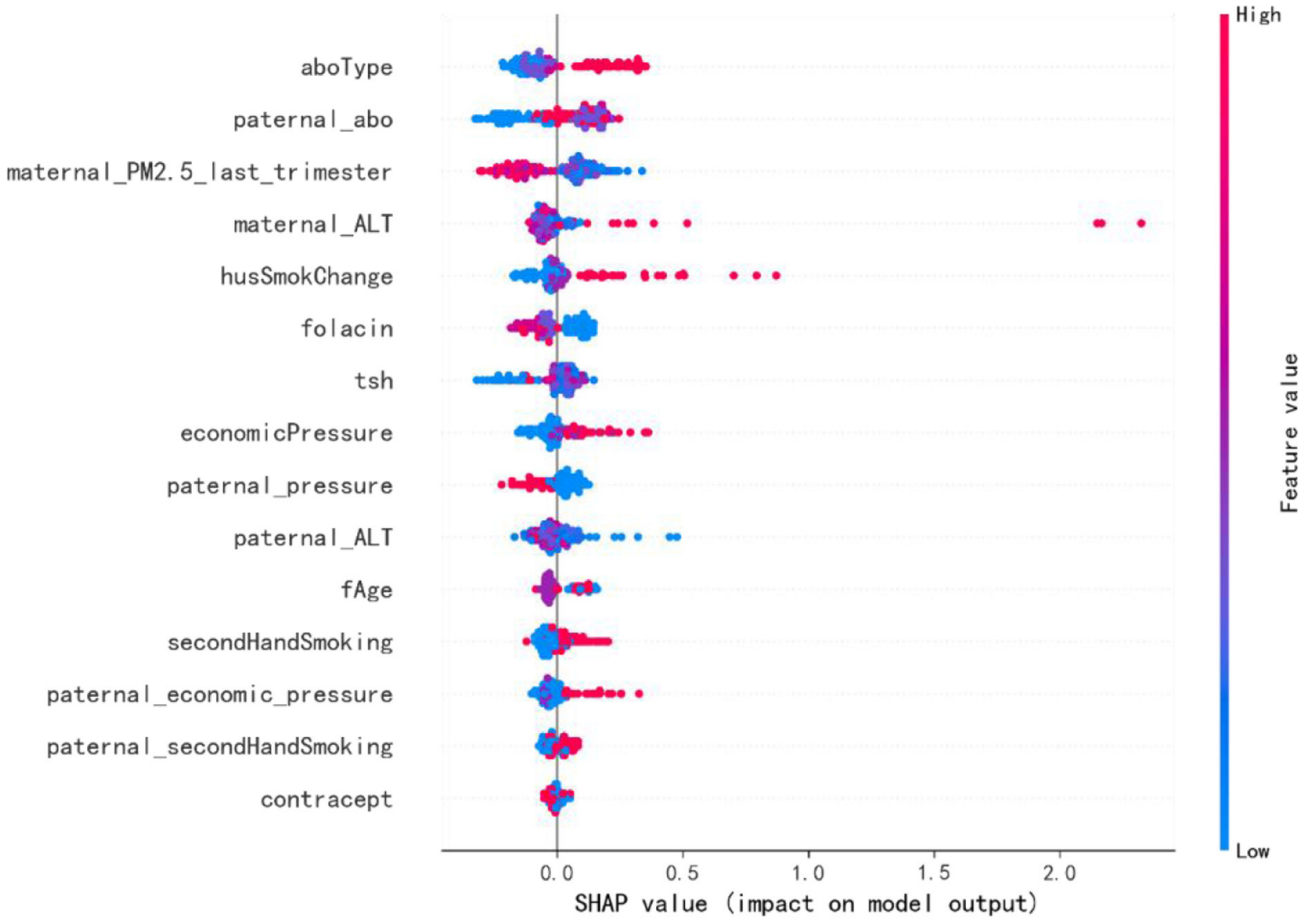
The Shapley Additive Explanation (SHAP) values for the most important predictors of SGA in the final model. The abscissa is the SHAP value, which shows the degree of impact on the outcome. Each dot represents a case. If the feature's value is high (low), the plot is colored red (blue). ALT, alanine aminotransferase; TSH, thyroid stimulating hormone.

The final prediction model's SHAP graphic based on CatBoost algorithm was drawn to determine the variables with the most impact on the outcome (Figure 4). The importance of the features was shown on the y-axis from top to bottom, while mean SHAP values were exhibited on the x-axis. Each dot was a sample. If the variable had a high (low) value, the plot was highlighted in red (blue). The 5 most critical variables were maternal blood type, paternal blood type, maternal exposure to PM2.5 during the late pregnancy, maternal ALT prior to pregnancy, and paternal smoking status in the first trimester. The mean SHAP value of each feature in the final prediction model is shown in Supplementary Figure S2.

## Discussion

This is the first research to apply ML classifiers in the establishment and testing of tools for SGA prediction in mothers who were exposed to pesticides prior to pregnancy. Paternal features and maternal pregnancy exposure to PM2.5 were also creatively incorporated as prediction features in our ML models. The results demonstrate that in comparison to the traditional LR method, ML classifiers can generate more accurate prediction models, with the CatBoost model showing the top SGA prediction performance (AUC: 0.855), indicating the use of ML classifiers to predict SGA is a viable method. The ML prediction model might be used to provide a prenatal prediction of SGA neonates in order to improve perinatal outcomes and effectively manage at-risk pregnancies.

To safeguard crops in the agricultural planting region, pesticides are extensively utilized. Pesticide usage is estimated to be over 3.5 million tons per year globally, with China using 1.8 million tons of pesticides in 2015, making it one of the countries with the highest overall pesticide usage (46). Among them, common pesticide types include organophosphorus pesticides, carbamate pesticides and organochlorine pesticides (46, 47). With increased exposure to pesticides, the percentage of SGA newborns rose. Prenatal exposure to pesticides, such as 4-nitrophenol, organochlorine, organophosphate, and pyrethroid pesticides, could impair fetal anthropometric development and reduce birth weight, resulting in an increased risk of delivering SGA neonates (10–12, 48, 49). Pesticides have been found in amniotic fluid, demonstrating they may pass the placenta and cause non-negligible fetal exposures (50–52). Fetuses could appear to be more susceptible to pesticide residues than adults because of their rapid growth, growing organ systems, and immature metabolic pathways (13). Specifically, organochlorine pesticides have been linked to growth retardation due to their genotoxic, immunotoxic, endocrine disrupting, cytotoxic, and fetotoxic effects (53). Through disrupting glyceraldehyde, organophosphorus pesticides could have a negative impact on birth weight (54). However, to the best of our knowledge, no prediction tool has been established for SGA neonates in mothers who were exposed to pesticides prior to pregnancy. We compared seven ML classifiers' performances for SGA (Table 2). CatBoost algorithm achieved the greatest AUC value (0.855) among these models, followed by GBDT, XGBoost, RF, and LGBM. But with an AUC of 0.691, the LR model got the lowest AUC. This might be as a result of the LR approach's sensitivity to outliers and need for a big dataset in order to function well. Furthermore, LR model's performance might be harmed by the unbalanced dataset. Our findings demonstrated that in comparison to traditional LR method, the ML algorithms were more effective in predicting SGA neonates in women who had been exposed to pesticides prior to pregnancy (AUC: 0.855 vs. 0.691).

The prediction of SGA newborns held considerable promise with the help of advanced ML classifiers. The cause behind it was that traditional modeling approaches failed to discover interactions between predictors that may occur. The advanced ML algorithms explored in our present work might resolve such problems. The automated handling of multidimensional and multivariable data by ML algorithms might uncover unique correlations between certain features and SGA outcomes, as well as discover trends that would otherwise be ignored by researchers (55). In addition, a robust SGA prediction model (AUC: 0.811, Figure 3) could be established using only 15 features, including parental demographic features, accessible medical test indices, and local PM2.5 exposure, suggesting suitable variables were chosen from 142 candidate variables using the RFE method. The RFE approach is a backward elimination technique based on wrappers that recursively computes the learning function to generate a recursive ranking of a given feature set (56). The efficacy of the RFE algorithm has been fully confirmed in a variety of medical studies (32–35, 57, 58).

Although there is evidence of familial influence in the cause of SGA births, the majority of studies have focused on maternal factors. Paternal factors, which can also potentially predict adverse birth outcomes, have received far less attention. In fact, several paternal determinants, including paternal age, height, race, level of education, and smoking status, are proven to be risk factors for SGA births (23, 24, 59–62). Furthermore, high-level ambient PM2.5 has been associated with an increased risk of SGA, proving the adverse effect of ambient PM2.5 on fetal growth (25, 26). These features, however, were not taken into account in prior SGA prediction tools developed in the overall population. We found that paternal blood type, smoking status, life/work stress, ALT, economic pressure, secondhand smoking status, and maternal exposure to PM2.5 during the late pregnancy were all among the 15 most important variables, implying that paternal factors and pregnant women's exposure to PM2.5 played an important role in SGA newborns prediction in the study subjects.

SHAP method was applied to explain the influence of the selected features on the outcomes of prediction models. The SHAP values reflected the influence distribution of each variable on the model outcome (Figure 4). For example, a high maternal ALT level increased the risk of SGA newborns. A similar pattern was shown by the variables maternal economic pressure and paternal economic pressure. Also, A lower level of maternal TSH before pregnancy decreased the risk of SGA. On the contrary, a lower level of maternal PM2.5 exposure in the last trimester was associated with an increased risk of SGA. Besides, paternal continued smoking during pregnancy, maternal secondhand smoking before pregnancy, and paternal secondhand smoking before pregnancy were also associated with an increased risk of SGA newborns. Maternal blood type O was related with an increased risk of SGA. Blood type O has been reported to be an independent risk factor for preeclampsia and gestational diabetes mellitus, which may explain the relationship between maternal blood type and SGA (63, 64). Also, reduced fetal growth has been related to increased maternal ALT level in recent studies (65). Additionally, both maternal and paternal smoking have been considered to be linked to an increased risk of delivering SGA newborns (66–68), which is consistent with our results. Chronic fetal hypoxia caused by smoking as well as placental vasoconstriction and increased apoptosis of placental syncytiotrophoblasts caused by nicotine have been proposed as the hypothesized mechanisms (69, 70). A higher maternal TSH concentration has also been proven to be associated with a lower birth weight (71). Moreover, lower income and less-privileged social class have been associated with higher risk of having SGA births because of the fact that fetal development could be affected by maternal emotional and psychological environment (72, 73). However, the association between maternal PM2.5 exposure and SGA contradicts previous studies, which may be caused by the difference of PM2.5 concentrations. The average PM2.5 concentration in previous studies from developed countries ranged from 1.82 to 22.11 μg/m^3^, which is less than one-third of the mean level of our study (74).

There are several limitations in this study. Firstly, despite the fact that the data was obtained nationally, the sample size was small, which might imply bias. Secondly, the dataset lacked information on the type of pesticides and average daily exposure in mothers' living or working environment prior to pregnancy. Besides, data lacked information on the ultrasonic biometric measurements. Including these data in the prediction model might increase the model's accuracy and applicability. To better understand the real value of the ML model in predicting SGA neonates, the ML prediction model still has to be tested and used in actual clinical settings.

## Conclusions

In this work, seven ML algorithms were used to build prediction models for SGA neonates in mothers exposed to pesticides prior to pregnancy. The results suggest that ML algorithms perform well in the classification of SGA neonates. Using feature selection and optimization approaches, the final prediction model using the CatBoost algorithm shows good performance on the prediction for SGA (AUC: 0.811) solely utilizing 15 variables, including parental demographic features, accessible medical test indices, and local PM2.5 exposure. Moreover, SHAP analysis enhanced the interpretation of the impact of the chosen variables to the categorization of SGA neonates, complementing the prediction findings. In high-risk populations, the prediction model based on ML algorithms might be a potentially effective tool for predicting the delivery of SGA neonates.

## Data availability statement

The raw data supporting the conclusions of this article will be made available by the authors, without undue reservation.

## Ethics statement

The studies involving human participants were reviewed and approved by the Institutional Review Board of the National Research Institute for Family Planning, Beijing, China (protocol code 2017101702). Written informed consent to participate in this study was provided by the participants' legal guardian/next of kin.

## Author contributions

Conceptualization: XB, SC, and HP. Methodology: XB, MS, LY, KL, and HY. Software: XB and ZZ. Validation: XB and YL. Resources, supervision, and funding acquisition: HP. Data curation: XB and SC. Writing—original draft preparation: XB. Writing—review and editing: SC and HP. Visualization: HZ. Project administration: XB and HP. All authors have read and agreed to the published version of the manuscript.

## Funding

This work was funded by the Chinese Academy of Medical Sciences Innovation Fund for Medical Sciences (2021-I2M-1-023).

## Conflict of interest

Authors MS, YL, LY, and KL were employed by DHC Mediway Technology Co. Ltd. The remaining authors declare that the research was conducted in the absence of any commercial or financial relationships that could be construed as a potential conflict of interest.

## Publisher's note

All claims expressed in this article are solely those of the authors and do not necessarily represent those of their affiliated organizations, or those of the publisher, the editors and the reviewers. Any product that may be evaluated in this article, or claim that may be made by its manufacturer, is not guaranteed or endorsed by the publisher.

## Supplementary material

Supplementary Figure 1 Decision curve analysis (DCA) of the three prediction models based on CatBoost, GBDT and XGBoost after feature selection. X-axis indicates the threshold probability for SGA outcome and Y-axis indicates the net benefit.

Supplementary Figure 2 The mean Shapley Additive Explanation (SHAP) value of each feature in the final prediction model based on CatBoost.

Supplementary Figure 3 Receiver operating characteristic (ROC) curves of the model using GBDT algorithm generated after recursive feature elimination (RFE) in predicting SGA.

Supplementary Figure 4 The Shapley Additive Explanation (SHAP) values for the most important predictors of SGA in the GBDT model.

Supplementary Figure 5 Receiver operating characteristic (ROC) curves of the model using XGBoost algorithm generated after recursive feature elimination (RFE) in predicting SGA.

Supplementary Figure 6 The Shapley Additive Explanation (SHAP) values for the most important predictors of SGA in the XGBoost model.

Supplementary Table 1 One hundred forty-two features included in machine learning models as candidate features for predictors.
