# Supplementary figures and images for: Predictive models for small-for-gestational-age births in women exposed to pesticides before pregnancy based on multiple machine learning algorithms
Source: Front Public Health. 2022 Aug 8;10:940182. doi: 10.3389/fpubh.2022.940182 (PMC9394741; doi:10.3389/fpubh.2022.940182)

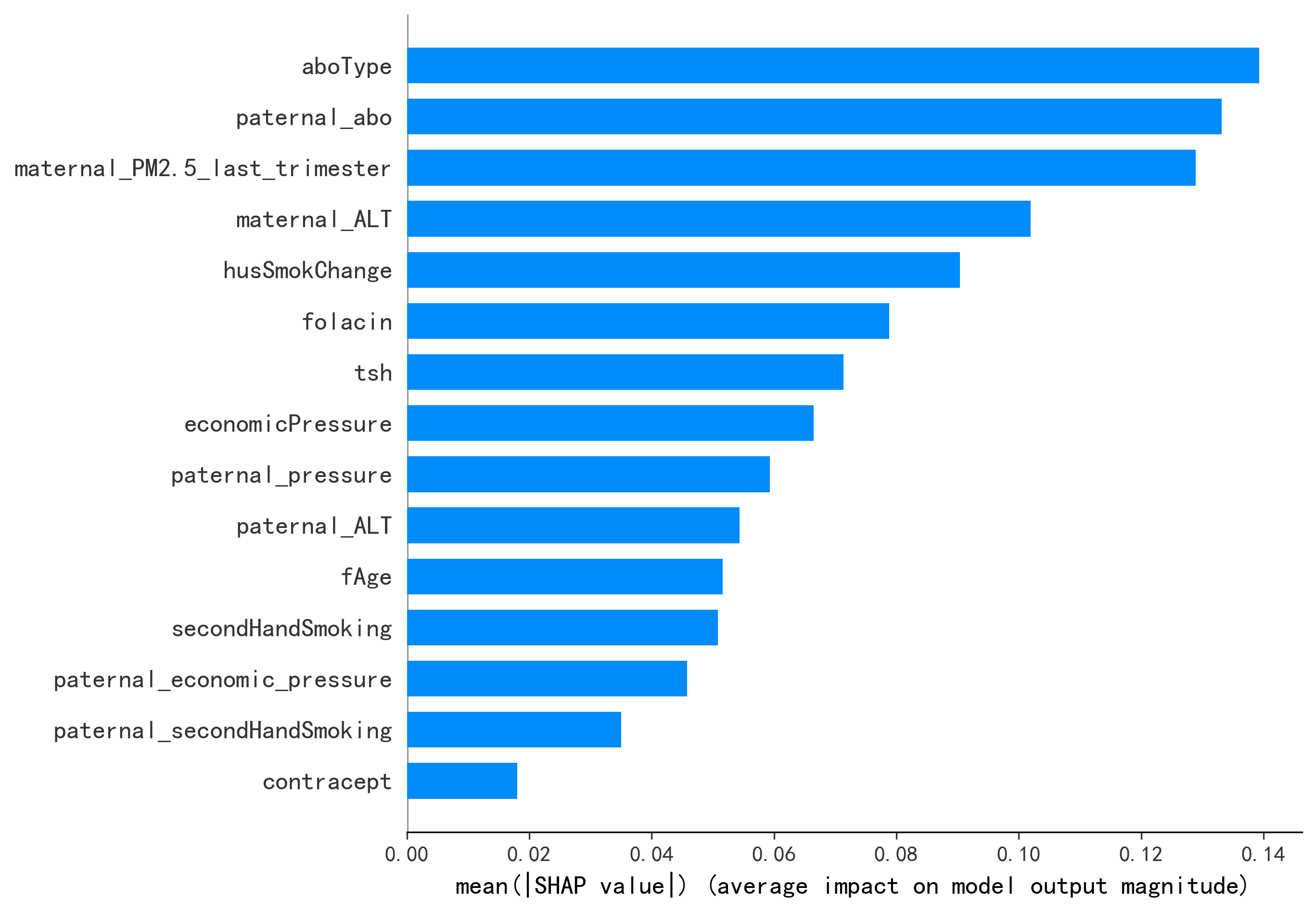

Supplement: Supplementary Figure 1 — Decision curve analysis (DCA) of the three prediction models based on CatBoost, GBDT and XGBoost after feature selection. X-axis indicates the threshold probability for SGA outcome and Y-axis indicates the net benefit. [file Data_Sheet_1.ZIP › Supplementary Figure 2.tiff]

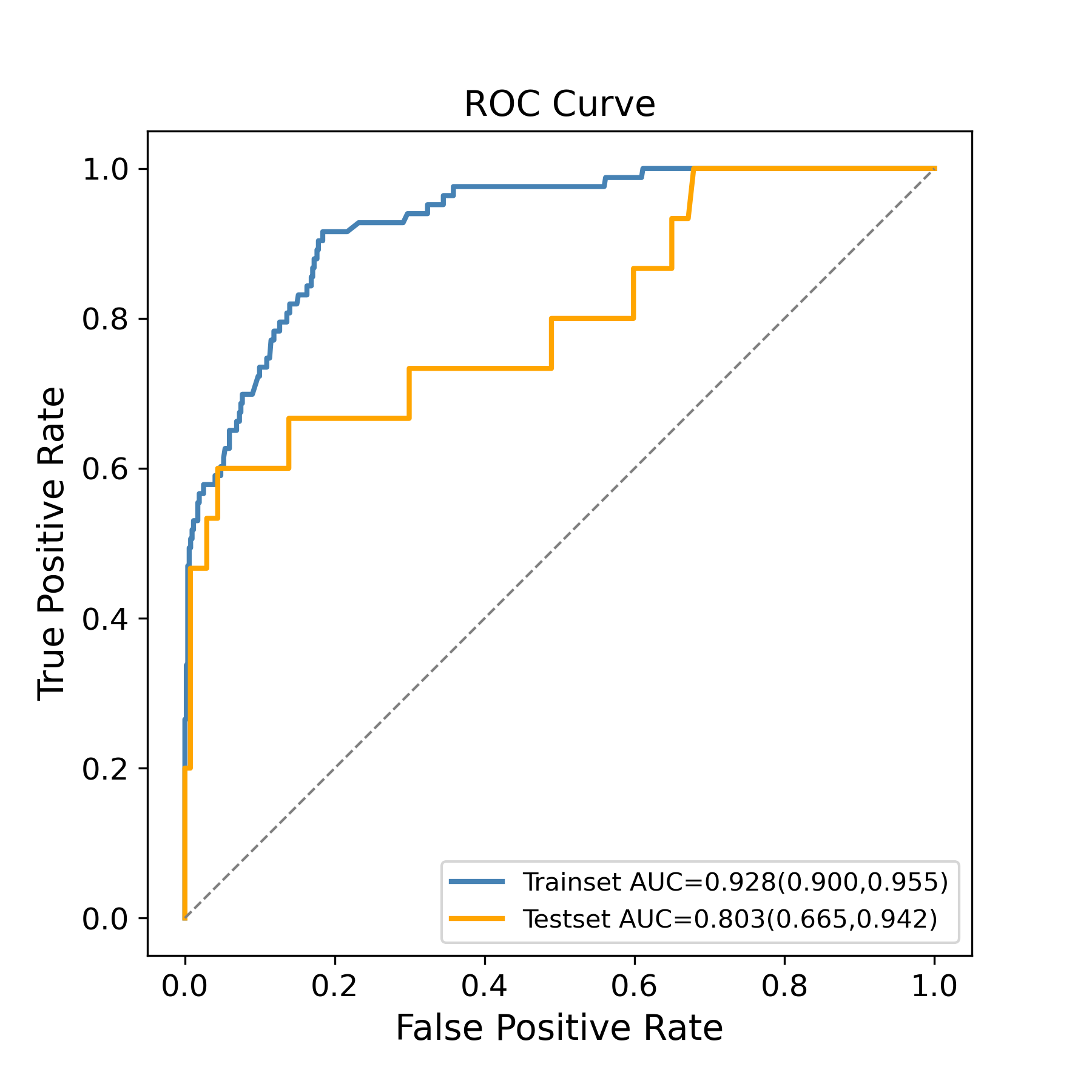

Supplement: Supplementary Figure 1 — Decision curve analysis (DCA) of the three prediction models based on CatBoost, GBDT and XGBoost after feature selection. X-axis indicates the threshold probability for SGA outcome and Y-axis indicates the net benefit. [file Data_Sheet_1.ZIP › Supplementary Figure 3.tiff]

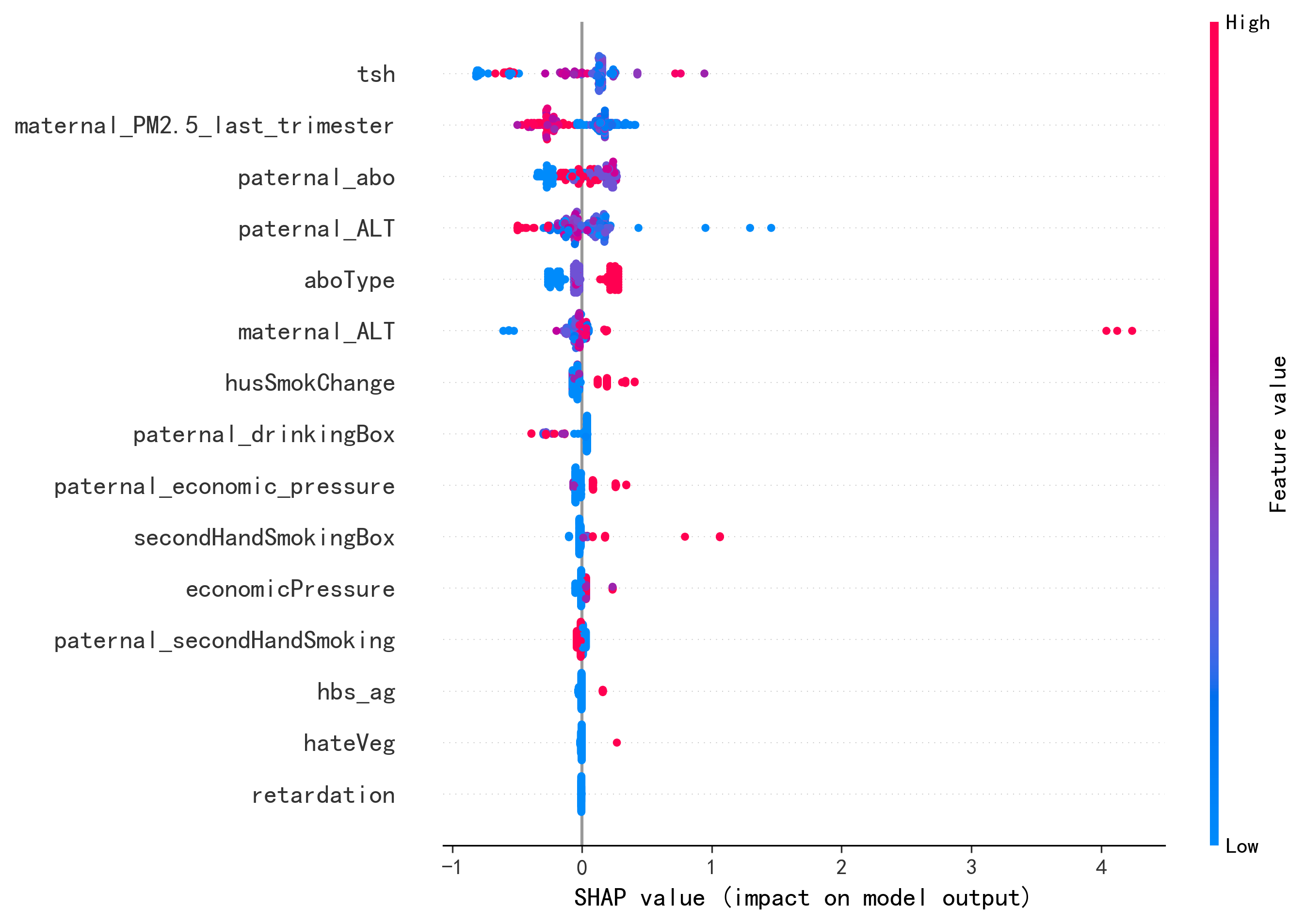

Supplement: Supplementary Figure 1 — Decision curve analysis (DCA) of the three prediction models based on CatBoost, GBDT and XGBoost after feature selection. X-axis indicates the threshold probability for SGA outcome and Y-axis indicates the net benefit. [file Data_Sheet_1.ZIP › Supplementary Figure 4.tiff]

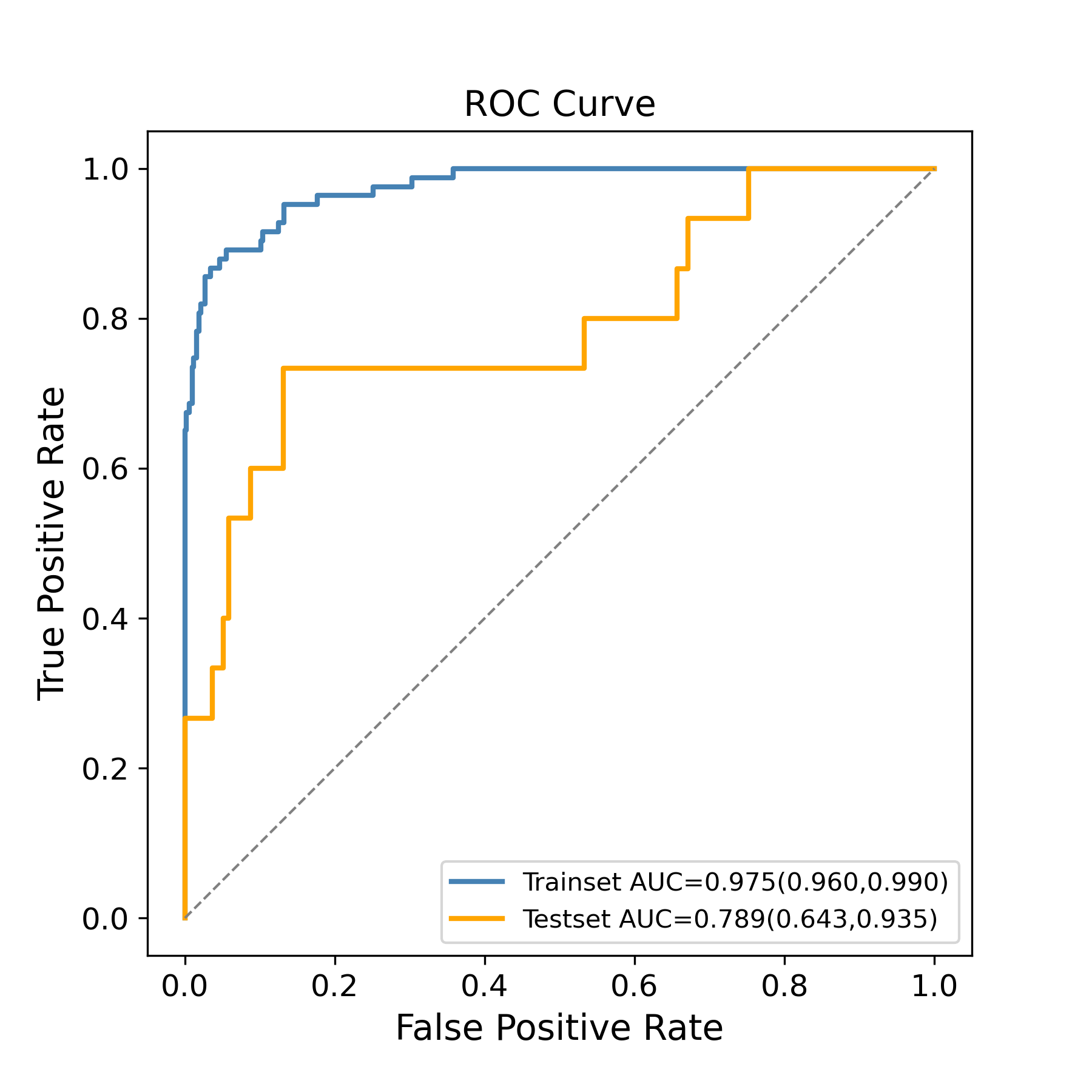

Supplement: Supplementary Figure 1 — Decision curve analysis (DCA) of the three prediction models based on CatBoost, GBDT and XGBoost after feature selection. X-axis indicates the threshold probability for SGA outcome and Y-axis indicates the net benefit. [file Data_Sheet_1.ZIP › Supplementary Figure 5.tiff]

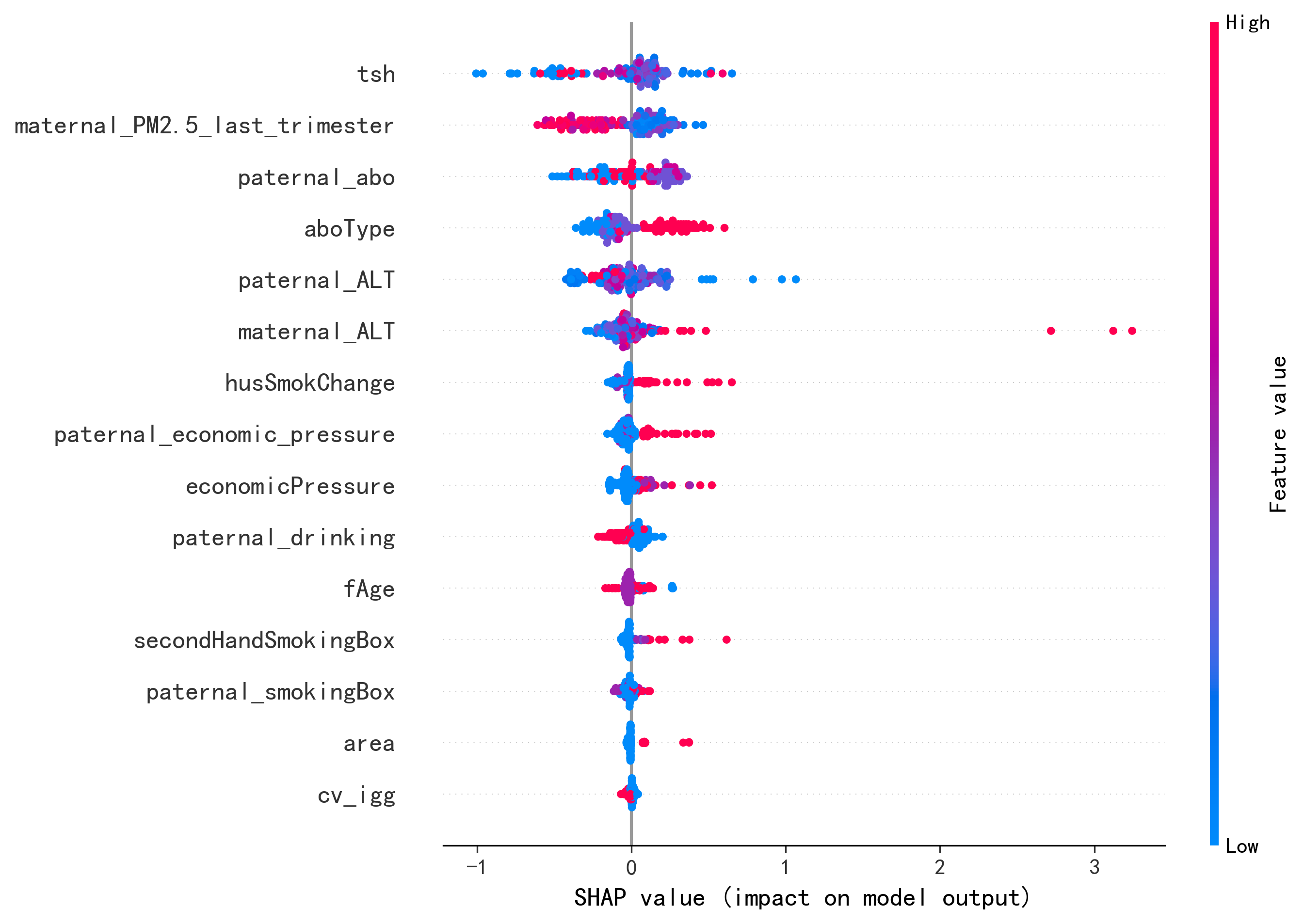

Supplement: Supplementary Figure 1 — Decision curve analysis (DCA) of the three prediction models based on CatBoost, GBDT and XGBoost after feature selection. X-axis indicates the threshold probability for SGA outcome and Y-axis indicates the net benefit. [file Data_Sheet_1.ZIP › Supplementary Figure 6.tiff]

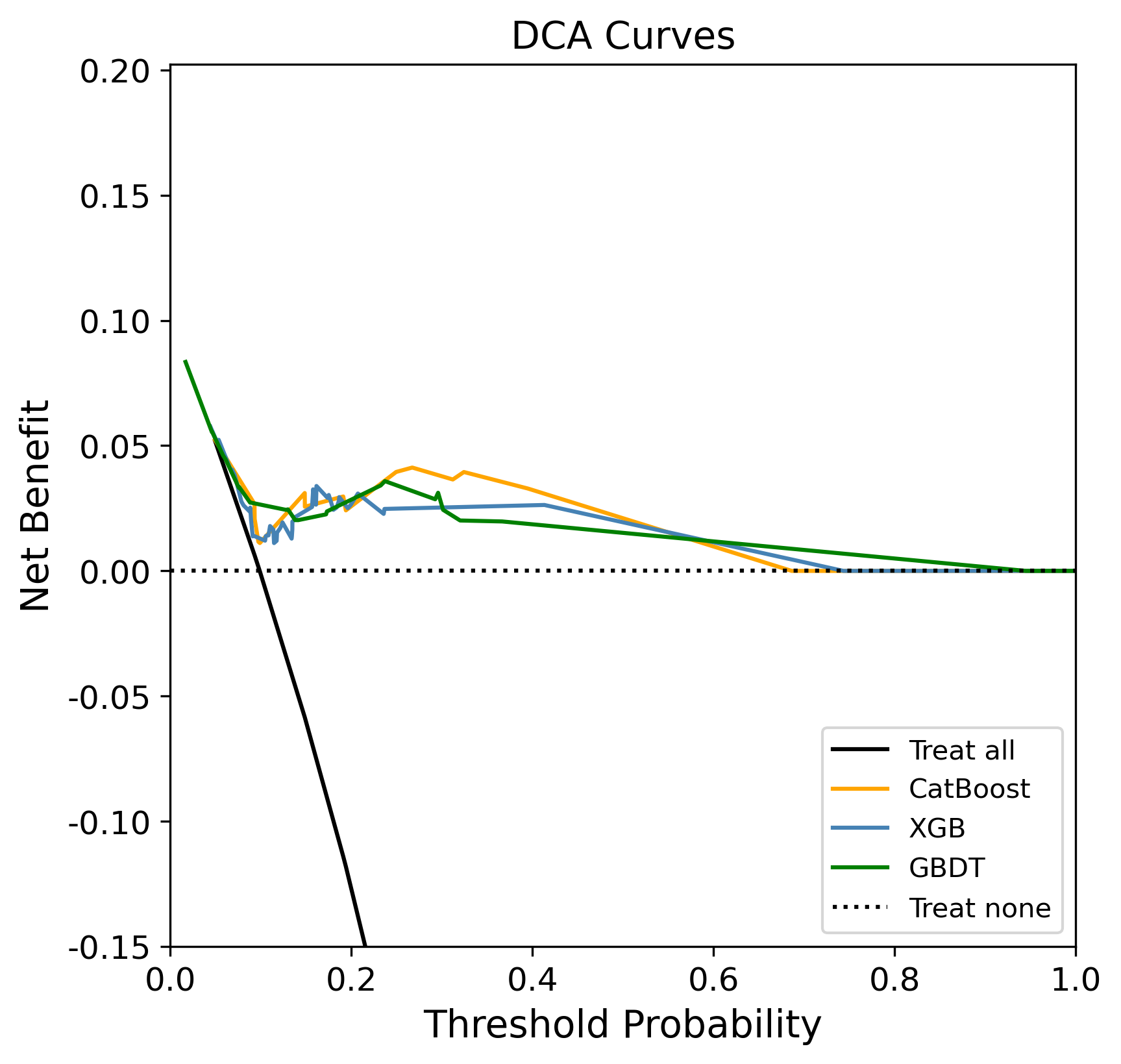

Supplement: Supplementary Figure 1 — Decision curve analysis (DCA) of the three prediction models based on CatBoost, GBDT and XGBoost after feature selection. X-axis indicates the threshold probability for SGA outcome and Y-axis indicates the net benefit. [file Data_Sheet_1.ZIP › Supplementary Figure 1.tiff]
